# Correction: Sexual orientation disclosure and depression among Thai gay, bisexual, and other men who have sex with men: The roles of social support and intimate partner violence

**DOI:** 10.1371/journal.pone.0307563

**Published:** 2024-07-18

**Authors:** Eduardo Encina, Worawalan Waratworawan, Yamol Kongjareon, Mayur M. Desai, Thomas E. Guadamuz

There are errors in the author affiliations. The correct affiliations are as follows:

Eduardo Encina^1,2^, Worawalan Waratworawan^1,3^, Yamol Kongjareon^1^, Mayur M. Desai^2^, Thomas E. Guadamuz^1,3,4^

**1** Center of Excellence in Research on Gender, Sexuality and Health, Faculty of Social Sciences and Humanities, Mahidol University, Nakhon Pathom, Thailand, **2** Department of Epidemiology of Microbial Diseases, Yale School of Public Health, New Haven, CT, United States of America, **3** Department of Society and Health, Faculty of Social Sciences and Humanities, Mahidol University, Nakhon Pathom, Thailand, **4** John F. Kennedy School of Government, Harvard University, Cambridge, MA, United States of America.
