# Supplementary material for: Sexual orientation disclosure and depression among Thai gay, bisexual, and other men who have sex with men: The roles of social support and intimate partner violence
Source: PLoS One. 2023 Nov 22;18(11):e0294496. doi: 10.1371/journal.pone.0294496 (PMC10664870; doi:10.1371/journal.pone.0294496)
Supplement: Attachment — Submitted filename: Responses to Reviewers_TG.docx [file pone.0294496.s001.docx]

Sexual Orientation Disclosure and Depression among Thai Men Who Have Sex with Men: Associations with Intimate Partner Violence and Social Support

Eduardo Encina, Worawalan Waratworawan, Yamol Kongjareon, Mayur M. Desai, Thomas E. Guadamuz

Journal: PLOS ONE

Reviewer's Responses to Questions/Comments

**We thank the reviewers for their constructive and insightful comments and suggestions. Below please find a point-by-point response to reviewers’ comments and suggestions. We feel that the manuscript has greatly benefited by the comments and suggestions.**

**Reviewer #1**: Strengths
There is a great need for research on the mental health of LGBTQ communities, particularly in non-Western societies. Furthermore, understanding the relationship of depression and intimate partner violence is of great significance for global public health. As such, this research is critically important. The purpose of the study has merit and has the potential to contribute to the field.

Areas of Weaknesses
The article being reviewed is an obvious contribution to the field. The study’s focus on depression, IPV, and sexual orientation disclosure among Thai men and is important and there is a dearth of research in this area. However, the manuscript as currently written is not suitable for publication. I would recommend accepting with several major revisions needed. Below are some tips to improve the quality of the manuscript for future publication.

Introduction
The introduction of the paper is very disjointed and does not underscore the rationale for the study. Specifically, it is unclear how sexual orientation disclosure relates to depression and IPV. The authors need to review the introduction to clearly tell the reader why they feel the relationship exist and its importance.

- Response: The introduction of the paper has been revised to properly reflect the relationships between disclosure, depression and IPV to reinforce the importance of this study (Introduction, lines 62-68, pages 3-4).

There are several places where the authors make claims that aren’t defended by either a citation or example of the existing research in the area (see lines 53, 57, 64, 65). Adding examples of the literature and citations for the claims would strengthen the arguments.

- Response: We have now included examples and provided proper citations (Introduction, lines 52-53, 56-57, 62-66, pages 3-4).

The aim of the study is to examine sexual orientation disclosure of gay and bisexual men however the authors use the term MSM to refer to the study population which is a behavioral term. Since the authors are referring to identity, the authors should use gay and bisexual not MSM which include people across sexual orientations. Furthermore, the authors should be consistent in language. Currently the authors use sexual minority, LGBTQ, and gay community to refer to the population. Please be specific and use consistent and correct language. On line 66, the authors use the term “surrounding spaces” but it is unclear what they are referring to. Please clarify.

- Response: We have now made the terms clearer and consistent throughout the manuscript. We feel that the term MSM is appropriate in the spaces where it is used because some participants identify as “straight” and not as “gay” or “bi.”
- Additionally, “surrounding spaces” was meant to refer to research on surrounding spaces for gay men. It has now been edited for clarity (Introduction, lines 68-71, page 4).

  Methodology issue

The authors should clarify what the Greater Mekong sub-region is for readers and identify the countries part of this region.

- Response: We have now clarified what the Greater Mekong sub-region is and the countries that are included in this region (Methods, lines 126-127, page 6).

The authors should identify the name of the study/project the data comes from.

- Response: This has been updated appropriately (Methods, lines 125-127, page 6).

It would be useful to the readers to have an example of one of the items parts of the IPV questions used in the study.

- Response: Example of the IPV questions such as "Hurting, hitting, slapping the body of a regular partner, casual partner or male sex work partner", "Forcing a regular partner or casual partner to have sex", "Forcing a person who is a male sex work partner to have sex." and "Fondling or unwanted sexual touching" have now been included in the Methods section (Methods, lines 187-189, page 9).

There is conflicting information about the age of participants. In one part of the paper, it states only persons over the age of 18 are included but in another it indicates people from 15-24. Please clarify who is included.
Analysis issues

- Response: We have checked and edited the manuscript so that all participants included in this study are age of 18 years and older.

The authors in the study state they had an initial sample of 1496 but only 1468 are included for analysis. The authors need to explain how they came up with this sample. Why were people excluded from the analysis?

- Response: In our final analysis, we decided to only include those individuals who fully answered all survey questions and clarified this appropriately in the new draft (Results, lines 235-236, page 11).

Discussion

A large issue with this paper is that the authors do not address this critical issue of what might impact sexual orientation disclosure or non-disclosure. The authors suggest disclosure is a good thing, however this omits the reality of how it might be harmful to disclose one’s sexual orientation. Furthermore, the authors suggest disclosure reduces depression however the analysis doesn’t allow from this level of analysis. You cannot determine direction based on this study. It could be that individuals who are depressed are less likely to disclosure their sexual orientation or that in fact disclosure leads to reduction of depression. The authors should address this limitation.

- The reviewer is correct that this study is limited by being a cross-sectional study and hence cannot establish causal relationships among the variables. We have now revised our discussion to reflect this limitation. (Discussion, pages 19 – 22).

On line 345 the authors incorrect use the term sexual orientation when they should be using gender identity.

- Response: This sentence was removed and edited in the new updated manuscript (Discussion, pages 19 – 22).
