# Supplementary material for: Sexual orientation disclosure and depression among Thai gay, bisexual, and other men who have sex with men: The roles of social support and intimate partner violence
Source: PLoS One. 2023 Nov 22;18(11):e0294496. doi: 10.1371/journal.pone.0294496 (PMC10664870; doi:10.1371/journal.pone.0294496)
Supplement: Attachment — Submitted filename: Response to Reviewers_TG.docx [file pone.0294496.s002.docx]

**Sexual Orientation Disclosure and Depression among Thai Men Who Have Sex with Men: Associations with Intimate Partner Violence and Social Support**

Eduardo Encina, Worawalan Waratworawan, Yamol Kongjareon, Mayur M. Desai, Thomas E. Guadamuz

Journal: PLOS ONE

**Reviewer's Responses to Questions/Comments**

**Title**
- Given the sample demographics, it might be more accurate and less misleading to use the term “gay, bisexual, and other men who have sex with men” throughout the manuscript or at least in the title instead of solely “MSM.”

**- Response: We agreed and already changed the title.**

**Abstract**
- “This article’s goal” should be reworded to sound less informal (e.g., “The purpose of this study,” “The goal of this study,” “The primary goal of this research,”)

**- Response: We agreed and already reworded to “The primary goal of this research”.**

- Should include that the majority of the sample identified as gay and bisexual. It cannot be assumed that the sample consists entirely of MSM participants unless you specify that in the Methods section of the abstract.

**- Response: We agreed and added gay, bisexual, and other men who have sex with men (GBM) to the methods section of the abstract.**

- The Discussion section of the abstract reads too much like the Results section. Rather than repeating findings, this section should synthesize and interpret major takeaways and highlight important implications. No implications are highlighted at the moment in this section.

**- Response: We agreed and have revised discussion on the abstract.**

**Introduction**
- The paper starts off by highlighting the experiences of LGBQ people specifically rather than MSM. I would recommend adding “questioning” to account for the small number of participants in your sample who may be questioning but still identify as heterosexual.

**- Response: We agreed and have included “questioning” to our list of LGBTQ.**

- Consider rewording the second sentence of the introduction to “Some key environmental and social influences that… include…”

**- Response: We have revised the sentence to “Some key environmental and social factors influence…”**

- Make sure the terms you use throughout the manuscript when citing studies are consistent with their samples (e.g., LGBTQ+, LGB, LGBQ, MSM).

**- Response: We have now made sure the terms we use throughout the manuscript when citing studies are consistent with their samples.**

- Could add subheadings to improve the organization of the introduction.

**- Response: We have now added subheadings.**

- Although the proposed associations are explained in the introduction, there does not appear to be an explicitly stated theoretical framework that guides the conceptualized models. For example, you reference “stressors” and “social stressors” a couple of times throughout the manuscript and cite research guided by minority stress theory, but do not mention minority stress theory or any other established theories that might help bolster your hypotheses.

**- Response: We have now re-structured to the Introduction to include a discussion on the minority stress theory and how it relates to the current study.**

- There are conceptual nuances to your proposed model that should be addressed in the Introduction section and/or the Discussion. As demonstrated by your findings and previous findings that you cite, sexual orientation disclosure is largely associated with positive mental health outcomes, but not always, as sexual orientation disclosure can lead to more frequent instances of discrimination, which can harm mental health/well-being. It is possible that greater outness may result in lower social support and a greater reliance on partners for support due to discrimination, which may exacerbate IPV experiences. Therefore, it should be emphasized that these processes are likely highly contingent upon the individual’s social environment and that lower levels of disclosure in certain contexts may be adaptive, as evidenced in the following studies:
1) van der Star, A., Pachankis, J. E., & Bränström, R. (2021). Country-level structural stigma, school-based and adulthood victimization, and life satisfaction among sexual minority adults: A life course approach. Journal of Youth and Adolescence, 50(1), 189–201. <https://doi.org/10.1007/s10964-020-01340-9>
2) Shepherd, B. F., Chang, C. J., Dyar, C., Brochu, P. M., Selby, E. A., & Feinstein, B. A. (2022). Out of the closet, but not out of the woods: The longitudinal associations between identity disclosure, discrimination, and nonsuicidal self-injury among sexual minoritized young adults. Psychology of Sexual Orientation and Gender Diversity. Advance online publication. <https://doi.org/10.1037/sgd0000597>

**- Response: We have now emphasized that these processes are likely highly contingent upon the individual’s social environment and that lower levels of disclosure in certain contexts may be adaptive. We have also included two studies you kindly provided for us.**

- Lastly, the hypotheses of your study need to be more clearly stated in the Introduction section. I recommend creating a “Present Study” or “Current Study” subsection to briefly state the study’s overarching purpose and specific hypotheses, especially given the complexity of the proposed multiple mediation model.

**- Response: We have included “Current Study” subsection to briefly state the study’s overarching purpose and specific hypotheses.**

**Methods**
- The Ns should be italicized in the tables and throughout the manuscript, as well as the p values.

**- Response: Ns have now been italicized in the tables and throughout the manuscript, as well as the p values.**

- Based on this statement (“Attenuation of the effect of disclosure on depression with the addition of the potential mediators was taken as evidence supporting the hypothesized mediation pathway”) and the results (e.g., no “indirect” or “direct” effects/associations were stated), I believe the authors may be confusing moderation with mediation. Attenuation alone is not sufficient evidence to support a mediation pathway, especially since the data are cross-sectional, but social support and IPV experiences may both affect the strength or directionality of the association between sexual orientation disclosure and emotional health as potential moderators. Please clarify or use alternative methodologies to test statistical mediation such as Hayes’ (2017) PROCESS macro. Was mediation hypothesized, but not tested?

**- Response:** **We have improved the methods section by including a clarification of the statistical mediators of interest.**

**Results**
- Subheadings are recommended to improve the organization of the Results section. There is a lot going on in this section and it is easy for readers to get lost, especially since a summary of the specific hypotheses tested was not provided in the Introduction section.

**- Response: We have now added subheadings in the results section.**

- I would avoid the use of the word “impact,” “led to,” and other words that may suggest causation between the variables of interest in the Results and Discussion sections, since the data are cross-sectional.

**- Response: We have now replaced “impact” with “association” throughout the manuscript.**

- In the tables, percentages of participants should be rounded so each column adds up to 100%.
**- Response: We have now rounded up percentages of participants so that each column adds up to 100%.**

**Discussion**
- The Discussion section has a lot of great information regarding sexual orientation disclosure, social support, IPV, and emotional health, but can be more strategically organized.

**- Response:** **We have now reorganized the discussion section as suggest.**

- The first paragraph is a summary of the study and your main findings, which is fine, but the following paragraphs read more like an Introduction section. In this section, each main finding should be explicitly stated, explained, and related to theories and prior research findings to highlight and support your contributions to the existing literature. You do this wonderfully in some paragraphs, but not so much in others.

**- Response: We agreed and have now made sure that the discussion section does not read like the introduction section and each main finding is explicitly stated, explained, and related to theories and prior research findings to highlight and support the study’s contributions to the existing literature.**

- I would recommend adding subheadings to improve the organization of the Discussion section. For example, you could create a “Limitations and Future Directions” section.

**- Response: We have now added subheading to improve the organization of the Discussion section.**

- The authors state that there might be “reverse causality” because of the cross-sectional design of their study. However, because the data are cross-sectional, causation cannot be inferred at all, in any direction, and that needs to be clearly stated. I recommend that the authors encourage future longitudinal research to address this limitation.

**- Response: We have included a statement to emphasize that causality cannot be inferred at all since this is a cross-sectional study. Longitudinal research to address this limitation urgently needed.**

Relatedly, the authors use too much causal/longitudinal language (e.g., “led to”) when discussing their findings, which again are based on cross-sectional data. Even the term mediator itself implies causation and should instead be called a “statistical mediator.” However, as previously stated, it is unclear whether the authors tested social support and IPV experiences as statistical mediators or jumped to conclusions based on independent associations/odds ratios.

**- Response: We have replaced the word mediators with statistical mediators throughout the manuscript. They are social support and IPV experiences.**
