# Supplementary material for: Sexual orientation disclosure and depression among Thai gay, bisexual, and other men who have sex with men: The roles of social support and intimate partner violence
Source: PLoS One. 2023 Nov 22;18(11):e0294496. doi: 10.1371/journal.pone.0294496 (PMC10664870; doi:10.1371/journal.pone.0294496)
Supplement: Attachment — Submitted filename: Response to Reviewers_Oct14.docx [file pone.0294496.s003.docx]

**Responses to Reviewers’ Questions/Comments**

Sexual Orientation Disclosure and Depression among Thai Men Who Have Sex with Men: Associations with Intimate Partner Violence and Social Support

Eduardo Encina, Worawalan Waratworawan, Yamol Kongjareon, Mayur M. Desai, Thomas E. Guadamuz

Journal: PLOS ONE

Dear editor and reviewers: We thank the editor and reviewers for your feedback, suggestions and the recommended readings. We have subsequently edited the paper according to the suggestions which we feel have greatly improved the quality of the paper. We have included a discussion on the importance of Social Support as an important variable in this paper in the Introduction and have adjusted the title. We also updated and adjusted the future directions and conclusion of the paper. Finally, we have edited the paper for better flow and had a native English speaker make final edits throughout the paper. Please find below a point-by-point response to the reviewers’ comments.

**Reviewer #1:** I appreciate the author's consideration of the reviewer's comments. I believe the current iteration is improved. I only have minor suggestions to improve the article.

***1.1 I would suggest the title indicate Mekong Region.***

Response: While the name of the study is “Greater Mekong - Young MSM Internet Survey”, only the Thai data was analyzed for this paper. We have now added the word “Thai” in the title to be clearer to the readers.

**1.2 The introduction is greatly improved. I would suggest the authors provide either a theoretical or empirical rationale for why social support is included.**

Response: We have now added to the Introduction an empirical rationale for why social support is included in the analysis.

**Reviewer #2**: I'm quite satisfied with the revisions that have been made based on the rounds of review and look forward to seeing the article published in PLOS One. My only remaining concern is the future directions section at the end of the paper.

**2.1 Research limitations are adequately described, but the real-life applications of your paper are not. For example, you briefly mention implications for public policy and community efforts/resources in the abstract and introduction, but do not clearly tie your findings to these implications in the discussion section even though implications for policy and community resources are critical to the purpose and theoretical basis for your paper (e.g., structural/community stigma leads to increased sexual orientation concealment and decreased community resources).**

Response: We have now added some real-life applications of the findings of our paper to the future directions section at the end of the paper.

**2.2 Implications for clinical practice (e.g., affirmative mental health conceptualizations/interventions) could also be described. Overall, your paper aims to increase awareness on ways to mitigate depressive mental health outcomes, but needs to provide readers (especially policy makers, community advocates, and mental health practitioners) with a better understanding of how this awareness can be translated into action.**

Response: We have now added implications to the discussion section of the paper.

**2.3 I also think the concluding sentence of the paper can be improved upon/reframed to end on a stronger note. Too many ideas are crammed into a single sentence and overgeneralizations are used. I would avoid calling the entire country "extremely tolerant and gay-friendly." If this was the case, sexual orientation disclosure would not be an issue. The word tolerant also implies that sexual diversity is something to be "tolerated" rather than accepted or embraced, so I would avoid using that language as well in academic writing.**

Response: We have now reframed the concluding sentence of the paper.
